# Acupoint Therapy on Diabetes Mellitus and Its Common Chronic Complications: A Review of Its Mechanisms

**DOI:** 10.1155/2018/3128378

**Published:** 2018-10-22

**Authors:** Yiyi Feng, Yuchen Fang, Yiqin Wang, Yiming Hao

**Affiliations:** Shanghai Key Laboratory of Health Identification and Assessment/Laboratory of TCM Four Diagnostic Information, Shanghai University of Traditional Chinese Medicine, Shanghai, 201203, China

## Abstract

Acupoint therapy is one of the therapeutic means in Traditional Chinese Medicine (TCM) concerning acupoints and meridians, including manual acupuncture, electroacupuncture, moxibustion, external application, acupoint injection, and catgut embedding. In the treatment of diabetes and its common chronic complications, acupoint therapy has proved to have specific curative effect and notable advantages. Single or combined with western medicine, it has superior efficacy and less side effects than western medicine alone. Studying its mechanism can provide experimental basis for clinical treatment. Relevant researches in the recent 5 years mainly focused on the mechanism of electroacupuncture, point injection, catgut embedding, etc. in the treatment of diabetes and common diabetic complications such as neuropathy, nephropathy, and hepatopathy. The possible theories involve the regulation of nerve conduction, signal pathways, hormone level, protein expression, oxidative stress level, structure restoration, etc. The most studied acupoints are Zusanli (ST36), Shenshu (BL23), Sanyinjiao (SP6), Yishu (EX-B3), and Zhongwan (CV12). However, most of the studies have been based on diabetes model rats rather than clinical trials. Moreover, the mechanism of acupoint therapy treating other chronic complications like diabetic retinopathy and that of other effective methods like pressing ear with beans, auricular points plaster therapy, and external application remain unclear. Therefore, this aspect still awaits further research.

## 1. Introduction

Diabetes mellitus (DM) is a ubiquitous metabolic disease which severely threatens the health and even the survival of humankind. Chronic hyperglycaemia is very likely to result in vascular damage, which will lead to a series of diabetic complications in heart, liver, stomach, kidney, muscle, peripheral nerve, etc. According to Global Report on Diabetes [1], diabetic complications may cause heart attack, stroke, blindness, renal failure, and lower limb amputation which have deadly consequences; in 2012, DM alone brought about the death of 1.5 million patients while its complications devitalized another 2.2 million patients. Acupoint therapy of Traditional Chinese Medicine (TCM) may be a way out. It is one of the therapeutic means in TCM concerning acupoints and meridians, including manual acupuncture, electroacupuncture, moxibustion, external application, acupoint injection, and catgut embedding. Acupoint therapy has proved to have specific curative effect and notable advantages in the treatment of DM and its chronic complications. Single or combined with Western medicine, it has more superior efficacy and less side effects than Western medicine alone. Many researches have shown that methods like electroacupuncture, acupoint injection, and catgut embedding can effectively decrease blood glucose, enhance insulin sensitivity, alleviate symptoms of various complications, and even prevent diabetes in specific populations [2]. Studying its mechanism will be conducive to its clinical application, thus benefiting diabetic patients worldwide. Our study reviewed relevant researches in the recent 5 years regarding the latest progress of the mechanism study of acupoint therapy in the treatment of DM and its common chronic complications. Currently, most of the studies are experiments on streptozotocin- (STZ-) induced Type 2 Diabetes Mellitus (T2DM) model rats.

## 2. The Mechanism Study of Hyperglycaemia Acupoint Treatment

Hyperglycaemia, which resulted from insulin secretion dysfunction or impairment of insulin biological function, is one of the most fundamental characteristics of diabetes mellitus. Acupoint therapy has been repeatedly confirmed to have evident effects on decreasing blood glucose. The existing studies have found that the regulation of related nerve excitement, protein expression, and signaling pathways that strengthens insulin sensitivity plays an important role in the treatment.

The endocrine system and the nervous system coregulate the metabolic activities in our bodies and they influence each other significantly. Yishu (EX-B3) is located below the 8^th^ spinous process of thoracic vertebrae, where there is part of T_8_ that innervates the pancreas. Electroacupuncture on T2DM rats at point Yishu (EX-B3) helps to reduce blood glucose and protect the shape of islets, which is consistent with the theory of innervation of neural segment [3]. The theory describes the neural connection between body surface and internal organs. The main functions of a certain acupoint are focused on the organs innervated by neural system adjacent to the acupoint. As long as a group of acupoints are located in the same neural segment, their functions are similar to each other even if they are not from the same meridian [4]. Obviously, the regulation of Yishu (EX-B3) on the pancreas is conformable to this theory. Besides, the hypothalamic-pituitary-adrenal axis (HPA), as an important part of neuro-endocrine system, also exerts influence on the insulin secretion. Electroacupuncture at Yishu (EX-B3) is demonstrated to decrease the level of adrenal cortical hormone (CORT) and reduce HPA hyperfunction, thus ameliorating endocrine dyscrasia and improving physical as well as psychological health status of rats to some extent [5].

Another important initial factor of T2DM is insulin resistance. One of the characteristic indexes of insulin resistance is high fasting insulin (FINS) level due to the patients' poor insulin sensitivity and low efficiency of uptaking and utilizing glucose. Electroacupuncture at Zusanli (ST36) and Shenshu (BL23) is reported to reduce the level of FINS and protect islet B cell morphology by enhancing mRNA expression of GLUT2 and GCK [6]. GLUT2 is an efficient carrier for glucose and GCK is an enzyme that facilitates phosphorylation of glucose, both of which function together as “glucose sensor” and respond to fluctuations of glucose level. Enhancement of them leads to improvement of insulin sensitivity and ameliorate insulin resistance. Manual acupuncture on Quchi (LI11), Hegu (LI4), Zusanli (ST36), Xuehai (SP10), Fenglong (ST40), Yinlingquan (SP9), Diji (SP8), Sanyinjiao (SP6), and Taichong (LR3) with “spleen-stomach harmonizing” technique can also reduce insulin resistance. “Spleen-stomach harmonizing” technique is created by Doctor Zhang Zhilong with a series of operations on the acupoints mentioned mainly for curing Type 2 diabetes mellitus and its complications. The study shows it possibly reduces insulin resistance by increasing the gene expression level of IRS-1, IRS-2, and GLUT4 in muscular tissue which suggests an activation of the signal transduction pathway of the phosphatidylinositol 3-kinase (PI3K)/Akt [7]. More studies have confirmed the effect of electroacupuncture on the PI3K/Akt signal transduction pathway. In this pathway, the activity of PI3K of insulin resistant rats is usually inhibited. Normally, its regulatory subunit p85 adjusts the catalytic activity of another subunit p110 and realizes the functions of insulin. However, exceedingly expressed p85*α* competitively inhibit the binding of p85-p110 and its downstream molecule IRS-1 so that the function of PI3K is suppressed. A study found that electroacupuncture at Zusanli (ST36) and Quchi (LI11) decreases the expression of PI3K-p85 to ameliorate the insulin resistance of model rats [8]. A more explicit research has shown that electroacupuncture at Neiguan (PC6), Zusanli (ST36), Sanyinjiao (SP6), and Shenshu (BL23) restores the level of insulin signaling related molecules like IRS-1, IRS-2, Akt2, aPKC*ζ*, and GLUT4 to normal and reversed the increased PI3K-p85*α* level [9]. In this way, metabolic activities like glycogen synthesis are improved via PI3K/Akt signaling pathway. In addition, the improvement of insulin resistance can reverse the pathological changes of vascular endothelium. Vascular endothelial dysfunction is the pathological basis and early stages of atherosclerosis, which is inseparable and forms a vicious cycle with the occurrence of insulin resistance [10]. It turns out to be another result of PI3K/Akt signalling pathway regulation. Researchers have studied the effect of electroacupuncture at Shenshu (BL23), Neiguan (PC6), Zusanli (ST36), and Sanyinjiao (SP6) on T2DM rats which demonstrates an increase in the expression of IRS-1, PI3K, Akt2, and eNOS in vascular endothelium. This signal transduction pathway is the downstream of insulin in vascular endothelium cells. When insulin sensitivity is enhanced, the signalling pathway can be better activated and promote the production of NO. In this way, vascular endothelial dysfunction is mitigated, and vasodilation can exert positive counteraction on glucose utilization [11].

## 3. The Mechanism Study of Diabetic Neuropathy Acupoint Treatment

Diabetic neuropathy is a kind of peripheral or central neuropathy caused by diabetic metabolic disturbance and angiopathy. The percent of complicated neuropathy ranges from 60% to 80% and its mortality rate reaches 20% in ten years from its occurrence [12]. It includes a wide variety of diseases and acupoint therapy interferes them in different mechanisms as follows.

### 3.1. Diabetic Peripheral Neuropathy (DPN)

The incidence of peripheral neuropathy in T2DM patients reached more than 60% and among them only approximately 53% of DPN patients survive after 3 years of its occurence [13, 14]. It may decrease sensory nerve conduction velocity (SNCV) and impair sensation or movement. Acupoint therapy can effectively increase SNCV and alleviate subjective symptoms. The mechanism involves upregulating the positive factors and downregulating the negative ones. For instance, electroacupuncture and acupoint injection with mecobalamin at Zusanli (ST36) and Shenshu (BL23) are demonstrated to increase the expression of nerve growth factor (NGF) and its receptor TrkA [15]. Likewise, catgut implantation at Pishu (BL20), Shenshu (BL23), and Zusanli (ST36) combined with taking herbal remedy, TCM prescription “Tangtong drink,” enhances the content of NGF plus lower that of serum transforming growth factor-*β*1 (TGF-*β*1) [16]. They both promote the regeneration of nerves and repair of nerve injuries. Other positive regulative factors that are reported to be raised by acupuncture or acupoint injection includes superoxide dismutase (SOD) expression [17], content of nitric oxide (NO) in the serum [18]. SOD is an important antioxidant defense of living cells exposed to oxygen and can have positive effect on alleviating cell damage. Low-level of NO, as previously mentioned, is related to vascular endothelium dysfunction that can lead to nerve hypoxia and ischemia. What is more, NO is an important inhibitory neurotransmitter and the lack of it can cause disorders of neural functions. The increase of these molecules apparently exerts benign effects on neuropathy patients. On the contrary, researchers who treated DPN patients with salvia injections at Zusanli (ST36) combined with lipoid acid intravenous injection found that the expression of high-sensitivity C-reactive protein (hs-CRP) and the content of malondialdehyde (MDA) were significantly decreased [17]. Another similar study on low-frequent electroacupuncture at Zhongwan (CV12), Mingmen (DU4), and Zusanli (ST36) combined with mecobalamin injection has shown the same result of reducing hs-CRP as well as homocysteine (Hcy) [19]. High level of hs-CRP, MDA, and Hcy mentioned above is correlated to causing or aggravating neuropathy, so their decrease protects the structure of neurons.

Another common DPN is diabetic gastroparesis. This disease usually results from vagus nerve lesion, which mainly manifests as delayed gastric emptying, decreased gastrointestinal motility and weakened gastric electrical activity with an incidence of 50%-76% [20]. Recent studies have focused on the interstitial cells of Cajal (ICCs) in the gastric antrum, gastrointestinal nervous system and hormones, etc. [21] Firstly, it was observed that the ultrastructure of ICCs and its pacing function was restored, and cell apoptosis remarkably reduced after the treatment of electroacupuncture at points of Zusanli (ST36) and Zhongwan (CV12). This is possibly concerned with the upregulation of stem cell factors (SCF) mRNA and neuronal nitric oxide synthase (nNOS) mRNA by mSCF/KIT-ETV1 signaling pathway [22–26]. Secondly, related hormones as important regulators of gastrointestinal functions and motility are affected directly or indirectly. For example, the levels of motilin, a gastrointestinal motility enhancer, and gastrin, the high-level of which has inhibitory effect on gastric movement, are obviously elevated in gastroparesis patients. The secretion of them will be inhibited by abdominal acupuncture at Zhongwan (CV12), Xiawan (RN10), Qihai (BL24), Guanyuan (BL26), Tianshu (ST25), Daheng (SP15), Huaroumen (ST24), and Wailing (ST26), as well as point injection of Vitamin B_12_ or mecobalamin at Zusanli (ST36), the effect of which is superior to using western medicine like mosapride alone [20, 27]. Studies of electroacupuncture at Zusanli (ST36), Liangmen (ST21), and Sanyinjiao (SP6) also revealed a growth in endothelial nitric oxide synthase (eNOS) mRNA, insulin, and its receptor (IR), as well as a cut-down on AT II mRNA, insulin-like growth factor-1 (IGF-1), and its receptor (IGF-1 R) in the gastric antrum. Among these molecules, the binding of insulin and IR protects ICCs and the expression of eNOS mRNA can lead to more abundant content of NO which can ameliorate vascular endothelial dysfunction as mentioned. AT II, on the other hand, play its role by binding with AT I receptor and AT II receptor, the former of which is found increased in gastric tissue of DGP rats. Likewise, IGF-1 and its receptor are found significantly higher in DGP rats so the cut-down of them might suggest an action mechanism of electroacupuncture [28–30]. As a matter of fact, the restoration of Cajal interstitial cells, mediation of hormones, and nervous system supplement each other. With the treatment of needling at specific points, the amount of Cajal interstitial cells increases and gastric slow wave is regulated, thus alleviating gastric myoelectric arrhythmias, promoting signal transduction between nervous system and smooth muscle, and eventually normalizing gastric motor [31]. However, there still remain some mysteries unsolved. Researchers have paid attention to the modulation of ghrelin and its role in strengthening gastric motility. Some concluded that electroapuncture at Zusanli, Liangmen (ST21), and Sanyinjiao (SP6) contributes to an increase in ghrelin and growth hormone secretagogue receptor (GHSR) mRNA while another group had a contradictive result under the similar experimental conditions that the level of ghrelin mRNA declines after the treatment [32–35]. Whether it is because of human error or an undiscovered underlying mechanism needs further investigation. One conjecture of our group is that some unknown factors made the pathology of diabetic gastroparesis of the model rats in two groups different. One decreases the ghrelin while the other increases it. Acupuncture played a mediative role, respectively, in both cases and had the tendency to balance the body status.

### 3.2. Diabetic Cardiac Autonomic Neuropathy

Diabetic cardiac autonomic neuropathy is the damage of structure or functions of cardiac autonomic nerves due to chronic hyperglycaemia. In a research studying the therapeutic effect of electroacupuncture at Feishu (BL13), Xinshu (BL15), Yishu (EX-B3), Pishu (BL20), Neiguan (PC6), Quchi (LI11), Zusanli (ST36), and Sanyinjiao (SP6), the inhibition of NGF excessive expression was observed and the content of ChAT, CNTF, and GAP-43 raised strikingly, the latter three of which participate in the restoration of nervous injuries [36].

NGF has the biological functions of nutrition and growth promotion of nerves. It is essential in maintaining normal growth and development of nerves, but its excessive expression may lead to abnormal proliferation of neurons. As discussed above, we know that NGF has low expression in the patients with diabetic peripheral neuropathy, and their NGF increases after electroacupuncture. An experiment of rats has also proved that NGF can promote peripheral nerve regeneration [37]. But on the contrary, NGF is high in diabetic cardiac autonomic neuropathy which is caused by the endogenous NGF produced in myocardium for the maintenance of neural integrity in several heart tissues. This finding in a dog experiment also pointed out that exogenously infused NGF can also protect against neural stunning of sympathetic cardiac innervation [38]. And the electroacupuncture may have played the role of exogenous NGF in depressing the excessive expression of endogenous NGF. The specific mechanisms of the effect of electroacupuncture treatment on this disease need to be further studied.

### 3.3. Diabetic Foot

A diabetic foot exhibits ulcers or gangrenes resulted from lower limb microcirculation disorder led by diabetic neuropathy and vascular structural damage. 6-Keto-PGF_1*α*_ and thromboxane B_2_ (TXB_2_) are one of the most common and effective biochemical factors of regulating thrombosis balance, and pathological changes might break the balance and cause various disorders of blood circulation. Researchers discovered that Vitamin B_1_ injection at point Jiexi (ST41) increases the content of 6-Keto-PGF_1*α*_ and decreases that of TXB_2_ to maintain the original balance in grade 0 diabetic foot patients. They reckoned that the acupoint injection boosts the nutrition supplement of neurons, restores part of its functions and exert feedback influence on blood circulation. In the progress, specific regulating factors of blood circulation like 6-Keto-PGF_1*α*_ and TXB_2_ are affected and regional microcirculation is ameliorated [39].

### 3.4. Diabetic Anterior Horn Injury of Spinal Cord

Diabetic anterior horn injury of spinal cord is another common nervous lesion. It usually implicates motor neurons of spinal cord, etc., and brings about muscular atrophy and a decline of muscular tension. A study on diabetic model rats demonstrated that the protein expression of apoptosis gene of anterior horn neurons of spinal cord, Bax, was remarkably lowered while its correspondent antiapoptosis gene Bcl-2 had a substantial increase after the treatment of electroacupuncture at Zusanli (ST36) and Yishu (EX-B3). As a result, the ratio of Bcl-2 and Bax was upregulated and was approaching normal standards [40]. This suggests that electroacupuncture can modulate gene expression and prevent the apoptosis of neurons.

### 3.5. Diabetic Myopathy

As mentioned above, neuropathy can lead to myopathy, a complication of great importance but often neglected. The mechanism of low-frequency electrical stimulation at Yanglingquan (GB34) and Zusanli (ST36) involves a variety of biochemical signals. First, it upregulates IGF-1 signaling pathway and the expression of microRNA myomiR which is related to muscle regeneration. This is followed by an increase in phosphorylation level of factors concerning protein synthesis like AKT, FoxO1, mTOR, and p70S6. Then, a series of pathological effects in diabetic model rats are reversed including the reduction of Pax7, MyoD, myogenin, and MHC-emb expression. From the macroscopic view, the ramifications above can be embodied as promoting muscle regeneration, strengthening muscular functions and attenuating myopathy [41].

To summarize, the study of some of nervous disease is very limited. For instance, only grade 0 diabetic foot was studied in the category of diabetic foot but that of far severer grade still have urgent needs of research. Nevertheless, the mechanisms of different kind of neuropathy are concentrated on gene, mRNA, or protein regulation of neuron growth or apoptosis. Future studies can draw on ideas and experiences of similar nervous diseases and enrich the theories.

## 4. The Mechanism Study of Diabetic Nephropathy Acupoint Treatment

Diabetic Nephropathy (DN) is glomerular sclerosis led by glucose metabolism disorders, accompanied by pathognomonic symptoms like abnormal urinal protein. DN is one of the most common microvascular complications of diabetes, with an incidence of 20%-40% [42]. It is also one of the major causes of death for diabetic patients. Diabetic nephropathy is reversible at early stages, so prompt therapy may help injured kidney recover. We collected the mechanism study of acupoint injection, catgut implantation combined with Chinese medicine, and “spleen-stomach harmonizing” needling. These therapeutic means affect the structure and functions of kidney through different chemical signals.

Shuxuening injection at Zusanli (ST36), Sanyinjiao (SP6), Fenglong (ST40), Yinlingquan (SP9), and Zhongshu (DU7) alternately of DN patients is proved to have certain curative effects. Shuxuening is an extract from ginkgo leaf, which contains flavanoids, phenols, alkaloids, etc. The injection significantly decreases the expression of vascular endothelial growth factor (VEGF), the rise of which will cause an increase in expansion of extracellular matrix and mesangial matrix of kidney, and therefore lead to glomerular sclerosis [43]. Catgut implantation at Shenshu (BL23), Huiyang (BL35), and Guanyuan (BL26) combined with taking TCM prescription “Tangtong drink” cuts down the content of TGF-*β*1 and IGF-1 in the serum of DN model rats at early stages [44]. The former one is the crucial cell factor in the mechanism of glomerular sclerosis while the latter participates in the alteration of glomerular hemodynamics, cell hypertrophy, etc. The therapies both downregulate the harmful growth of particular cell factors and promote the repair of renal structure. The possible ramifications of the regulation are the amelioration of glucose metabolism and renal hemodynamics, the correction of high filtration and high perfusion status of kidney, the reduction of growth and differentiation of renal cells, etc., all of which alleviate nephropathy and improve renal functions.

“Spleen-stomach harmonizing” needling also plays an important role in the clinical treatment of DN. Acupuncture at Zhongwan (CV12), Quchi (LI11), Hegu (LI4), Xuehai (SP10), Zusanli (ST36), and Yinlingquan (SP9) with this technique efficaciously improves glomerular filtration and reduce urinary albumin excretion rate of DN patients. Specialized study of it also found extensive regulation of the patients' bodies. During their study, a number of genes and signaling pathways were investigated. The study mentioned 5 signaling ways that are upregulated, including glutathione metabolism, the interactions of cell factor receptors, etc. The downregulated genes control altogether 40 signaling pathways, concerning type 1 diabetes mellitus, PPAR, etc. Among them, gene IFNG and the signaling pathways it regulates concerning T cell receptor (TCR), hypoxia inducible factor-1 (HIF-1), JAK-STAT signaling pathway, TGF-*β*, etc. stand out. Furthermore, spleen-stomach harmonizing needling inhibits the excessive expression of MCP-1. The combination of these effects modulates the amount and activity of T cell subset, thus restoring lymphocyte injury [45, 46]. Meanwhile, the needling decreases the oxidative stress level of DN patients and strengthens the capability of organism to scavenge free radicals [47]. All of the above delay or alleviate renal damage.

## 5. The Mechanism Study of Diabetic Hepatopathy Treatment

Liver is of great significance to glucose metabolism of human body. Long-term hyperglycaemia may harm the structure of liver, thus counter-reflecting on glucose metabolism and forming a vicious cycle. As diabetic hepatopathy has only been raising people's awareness in recent years, the mechanism study of its treatment is still limited. The theories at present indicate that electroacupuncture at Yishu (EX-B3) enhances the expression of gene and protein of glucagon-like peptide-1 (GLP-1) receptor, a glucose-dependent insulin secretion enhancer, in the liver cells as well as decreases alanine aminatransferase (ALT) and aspartate aminotransferase (AST), and the biomarkers of liver cell damage, in the serum [48, 49]. These alterations protect liver cells and ultimately ameliorate diabetic hepatopathy.

## 6. Conclusions

As the circumstance of diabetes grows increasingly severe all over the world, acupoint therapy, as one of the most important parts of Chinese medicine, plays an indispensable role in the treatment of diabetes and its complications.

**Figure 1:**
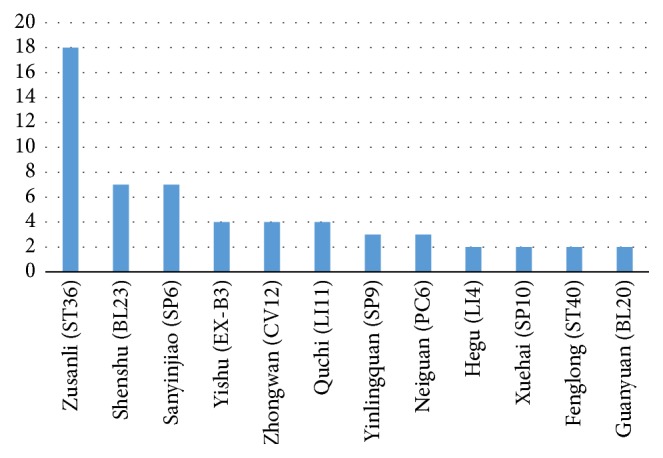
The appearance frequency of most mentioned acupoints in the articles of recent five years.

We listed the function and effect of acupoint therapy on DM and its common chronic complications in Table 1. From what we have collected, the mechanism study of treatment of hyperglycaemia and its common chronic complications like peripheral neuropathy and nephropathy is comparatively elaborate. However, the correspondent studies of diabetic foot, diabetic anterior horn injury of spinal cord, diabetic myopathy, diabetic hepatopathy, and other complications unmentioned above like diabetic retinopathy are shallow and rare. For instance, only grade 0 diabetic foot has been studied in the category of diabetic foot. The most studied methods of acupoint therapy are electroacupuncture, acupoint injection, catgut implantation, and manual acupuncture (usually with “spleen-stomach harmonizing” needling technique), but the mechanism of other effective ways like pressing ear with beans, auricular points plaster therapy, and external application remains unclear. As shown in Figure 1, the most studied acupoints are Zusanli (ST36), which is mentioned 18 times in all the articles we collected. This is followed by Shenshu (BL23) (7 times), Sanyinjiao (SP6) (7 times), Yishu (EX-B3) (4 times), Zhongwan (CV12) (4 times), and Quchi (LI11) (4 times). The rest of the acupoints are widely distributed. Still, the effects of different acupoints as well as different combination of them need more investigations and experiments. Moreover, most of the studies are experiments on model rats rather than clinical trials, so whether the results are consistent with human body awaits further research. Whether electroacupuncture can substitute manual acupuncture is another problem unsolved hitherto.

The mechanisms concluded involve the regulation of nerve conduction, signal pathways, hormone level, protein expression, oxidative stress level, and structure restoration. In biology, structures and functions are united and the abnormality of functions is usually concerned with structural damage. The treatment generally regulates some specific biochemical factors to influence particular signal pathways, eventually controlling the apoptosis and proliferation of cells and repairing the damaged structure. Sometimes the therapy has two-way regulation of one specific factor in different studies, and we speculate that the therapy is a process of balancing the whole body. Different pathology may cause rise or decline of the content of specific factors, and the therapy corrects them to go to the normal status. The studies usually focused on a few factors each time, but the therapy actually regulates the entire body. The nervous system, the endocrine system, the circulation system, etc. collaborate to achieve a more harmonious balance in human body. Due to the confined studies, it is basically impossible to draw a complete picture of the mechanism currently. Nevertheless, the present researches have laid the foundation and pointed out the possible directions. Future studies can draw on the experimental design and consideration of past researches, enrich the existing theories, and explore more on the clinical trials.

## Figures and Tables

**Table 1:** The function and effect of acupoint therapy on DM and its common chronic complications.

**Disease**	**Experimental object**	**Different acupoints combination**	**Acupoint therapy**	**Function**	**Effect**
Diabetes mellitus	T2DM rats	Yishu (EX-B3)	Electroacupuncture	1. Innervate the pancreas by neural segment 2. Reduce hyperfunction of hypothalamic-pituitary-adrenal axis	1. Protect the islets 2. Ameliorate endocrine dyscrasia
	T2DM rats	Zusanli (ST36), Shenshu (BL23)	Electroacupuncture	High-level expression of gene GLUT2 and GCK	Reduce insulin resistance
	T2DM rats	Quchi (LI11), Hegu (LI4), Zusanli (ST36), Xuehai (SP10), Fenglong (ST40), Yinlingquan (SP9), Diji (SP8), Sanyinjiao (SP6), Taichong (LR3)	Manual acupuncture	High-level expression of gene IRS-1, IRS-2, and GLUT4	
	T2DM rats	Zusanli (ST36), Quchi (LI11)	Electroacupuncture	Low-level expression of PI3K-85p	Reduce insulin resistance
	Otsuka Long-Evans Tokushima Fatty (OLETF) rats	Zusanli (ST36), Shenshu (BL23), Neiguan (PC6), Sanyinjiao (SP6)	Electroacupuncture	1. High-level of IRS-1, IRS-2, Akt2, aPKC*ζ*, and GLUT4 2. Low-level of PI3K-p85*α*	Reduce insulin resistance
	T2DM rats	Zusanli (ST36), Shenshu (BL23), Neiguan (PC6), Sanyinjiao (SP6)	Electroacupuncture	High-level expression of IRS-1, PI3K, Akt2, and eNOS	1. Reduce insulin resistance 2. Ameliorate vascular endothelial dysfunction

Diabetic peripheral neuropathy (include diabetic gastroparesis)	DPN rats	Zusanli (ST36), Shenshu (BL23)	1. Electroacupuncture 2. Acupoint injection of mecobalamin	High-level expression of nerve growth factor and its receptor TrkA	1. Promote the regeneration of nerves 2. Repair of nerve injuries
	DPN rats	Pishu (BL20), Shenshu (BL23), Zusanli (ST36)	1. Catgut implantation at acupoint 2. Take Chinese medicine	1. High-level expression of nerve growth factor 2. Low-level expression of transforming growth factor-*β*1	
	Chinese DPN patients	Zusanli (ST36)	1. Acupoint injection of salvia injections 2. Intravenous injection of lipoid acid	1. Low-level expression of high-sensitivity C-reactive protein 2. Low content of malondialdehyde 3. High-level expression of superoxide dismutase	Protect the structure of neurons
	Chinese DPN patients	Zusanli (ST36), Zhongwan (CV12), Mingmen (DU4)	1. Electroacupuncture 2. Acupoint injection of mecobalamin	1. Low-level expression of high-sensitivity C-reactive protein 2. Low-level expression of homocysteine	
	DGP rats	Zusanli (ST36), Zhongwan (CV12)	Electroacupuncture	High-level expression of stem cell factors and neuronal nitric oxide synthase	Restore the ultrastructure and pacing function of interstitial cells of Cajal in the gastric antrum
	Chinese DGP patients	Zhongwan (CV12), Xiawan (RN10), Qihai (BL24), Guanyuan (BL26), Tianshu (ST25), Daheng (SP15), Huaroumen (ST24), and Wailing (ST26)	1. Abdominal acupuncture 2. Acupoint injection of Vitamin B_12_	Enhance the secretion of motilin and gastrin	Regulate gastrointestinal functions and motility
	Chinese DGP patients	Zusanli (ST36)	Acupoint injection of mecobalamin		
	DGP rats	Zusanli (ST36), Liangmen (ST21), and Sanyinjiao (SP6)	Electroacupuncture	1. Growth in endothelial nitric oxide synthase, insulin and its receptor in the gastric antrum 2. Low content of AT II, insulin-like growth factor-1 and its receptor in the gastric antrum	1. Promote the restoration of Cajal interstitial cells 2. Mediate hormones and nervous system

Diabetic cardiac autonomic neuropathy	DM rats	Feishu (BL13), Xinshu (BL15), Yishu (EX-B3), Pishu (BL20), Neiguan (PC6), Quchi (LI11), Zusanli (ST36), Sanyinjiao (SP6)	Electroacupuncture	1. Low-level expression of nerve growth factor 2. High content of ChAT, CNTF, and GAP-43	1. Prohibit abnormal proliferation of neurons 2. Restore the injuries of nervous

Diabetic foot	Chinese level 0 diabetic foot patients	Jiexi (ST41)	Acupoint injection of Vitamin B1	1. High content of 6-Keto-PGF1*α* 2. Low content of thromboxane B_2_	1. Supply the nutrition of neurons 2. Ameliorate the regionalmicrocirculation of foot

Diabetic anterior horn injury of spinal cord	DM rats	Zusanli (ST36), Yishu (EX-B3)	Electroacupuncture	1. High-level expression of gene Bcl-2 2. Low-level expression of gene Bax	Prevent the apoptosis of neurons

Diabetic myopathy	DM rats	Yanglingquan (GB34) and Zusanli (ST36)	Electroacupuncture	1. Up-regulates IGF-1 signaling pathway 2. High-level expression of gene myomiR 3. Increase in phosphorylation level of factors concerning protein synthesis like AKT, FoxO1, mTOR, and p70S6 4. Low-level expression of Pax7, MyoD, myogenin and MHC-emb	1. Promote the muscle regeneration 2. Strengthen the muscular functions 3. Attenuating the myopathy

Diabetic nephropathy	Chinese DN patients	Zusanli (ST36), Sanyinjiao (SP6), Fenglong (ST40), Yinlingquan (SP9), and Zhongshu (DU7)	Acupoint injection of Shuxuening (extract from gingko leaf that contains flavanoids, phenols, alkaloids, etc.)	Low-level expression of vascular endothelial growth factor	1. Ameliorate the expansion of extracellular matrix and mesangial matrix of kidney 2. Ameliorate glomerular sclerosis
	DN rats	Shenshu (BL23), Huiyang (BL35), Guanyuan (BL26)	1. Catgut implantation at acupoint 2. Take Chinese medicine	Low content of transforming growth factor-*β*1 and insulin-like growth factor-1	1. Ameliorate the glucose metabolism and renal hemodynamics 2. Correct high filtration and high perfusion status of kidney 3. Reduce growth and differentiation of renal cells
	Chinese DN patients	Zhongwan (CV12), Quchi (LI11), Hegu (LI4), Xuehai (SP10), Zusanli (ST36), Yinlingquan (SP9)	Manual Acupuncture	1. Regulate gene IFNG, T cell receptor, hypoxia inducible factor-1, JAK-STAT signaling pathway, TGF-*β*, etc. 2. Low-level expression of MCP-1 3. Enhance the activity of superoxide dismutase	1. Improve glomerular filtration and reduce urinary albumin excretion rate 2. Modulate the amount and activity of T cell subset and restore lymphocyte injury 3. Improve the level of anti-oxidative stress

Diabetic hepatopathy	DM rats	Yishu (EX-B3)	Electroacupuncture	1. High-level expression of gene and protein of glucagon-like peptide-1 receptor 2. Low content of alanine aminotransferase and aspartate aminotransferase	Protect liver cells
